# Comparison of Mortality Rates Between Patients With Diffuse Large B Cell Lymphoma Surviving 5 Years After Initial Diagnosis and a Matched General Population Cohort

**DOI:** 10.1002/cam4.70800

**Published:** 2025-03-20

**Authors:** John Acquavella, Dóra Körmendiné Farkas, Henrik Toft Sørensen

**Affiliations:** ^1^ Department of Clinical Epidemiology Aarhus University Hospital and Aarhus University Aarhus Denmark

**Keywords:** cohort study, lymphoma, survival

## Abstract

**Aim:**

To determine whether mortality for patients with diffuse large B‐cell lymphoma who survived 5 years (DLBCL5ys) returns thereafter to general population levels.

**Methods:**

This population‐based cohort study included Danish residents between January 1, 2000, and December 31, 2023. Information on diagnoses, comorbidities, and vital status came from Danish health and administrative registries. Analyses included 4164 DLBCL5yr patients—55% of incident patients—and 41,640 individuals from the general population matched 10:1 by exact birth year, sex, and the calendar year of achieving DLBCL5yr status. We used Cox proportional hazards models to compute matched mortality hazard ratios (HRs) and 95% confidence intervals (CIs) and controlled for comorbidities by adding Charlson comorbidity index scores to our models.

**Results:**

Mortality rates were elevated for DLBCL5yr patients soon after their 5‐year survival date. The absolute difference in mortality was 20 deaths per 1000 person‐years, and the HR adjusted for comorbidities was 1.5 (95% CI 1.4–1.6). Mortality was elevated similarly for men and women. The elevated mortality for DLBCL5yr patients varied in magnitude by age, calendar period, and comorbidity burden, and included excess mortality from DLBCL, cancers other than lymphoma, and respiratory diseases.

**Conclusions:**

We found that mortality for DLBCL5yr patients did not return to general population levels.

## 1 Background & Introduction

Diffuse large B cell lymphoma (DLBCL) is the most common subtype of non‐Hodgkin's lymphoma and the fastest progressing B cell non‐Hodgkin's lymphoma [1]. However, although DLBCL tends to progress rapidly, it is considered potentially curable [1]. According to the US National Cancer Institute's Surveillance, Epidemiology, and End Results program, the 5‐year relative survival rate for patients with DLBCL increased from 37% in 1975 to 66% in 2005 [2]. This trend toward increased survival rates has been attributed to treatment advances, primarily the addition of rituximab to the cyclophosphamide, doxorubicin hydrochloride, oncovin, prednisone chemotherapy regimen (also known as CHOP‐R) [3]. Evidence has indicated modest continued increases in 5‐year survival rates through 2013, but new therapies or advances in earlier detection will probably be required to make further progress.

Five‐year survival after diagnosis is often considered a milestone for good prognosis for many cancers, which might portend survival, given other medical conditions, at rates similar to peers in the general population [4]. Comparative survival research in patients with DLBCL has focused largely on 2‐year survival, particularly for patients with 2‐year event‐free survival, or patients with 2‐year event‐free survival treated with specific chemo‐ or immunotherapeutic regimens [5, 6, 7, 8]. Results have been mixed regarding whether the survival of patients who are event‐free normalizes to that of the general population, but mortality has been found to be elevated for the total cohort of Swedish patients surviving 2 years after diagnosis [7]. We are not aware of any studies in the literature addressing whether patients with DLBCL who survive 5 years after the initial diagnosis (hereafter denoted DLBCL5yr patients) have mortality rates similar to those in the general population. Therefore, the primary purpose of this study was to compare all‐cause mortality rates between DLBCL5yr patients with that for age‐and sex‐matched general population peers. We also examined survival for all patients with DLBCL at 1, 3, and 5 years after their initial diagnosis during the study period.

## 2 Research Methods

### 2.1 DLBCL and Comparison Cohort Development

This nationwide cohort study in Denmark covered the period from January 1, 2000 to December 31, 2023. The Danish National Health Service provides universal tax‐supported health care for all Danish residents. The related data systems have been shown repeatedly to be of high quality for epidemiologic research [9].

A cohort of adult patients (≥ 18 years of age) diagnosed with DLBCL during 2000–2018 was established through the Danish Cancer Registry (DCR) by using the International Classification of Diseases, Tenth Revision (ICD‐10) code C83.3 and morphology codes 968.01, 968.03, and 968.09 [10]. The DCR, founded in 1942, includes information on the date of cancer diagnosis and tumor stage at diagnosis for all incident cancers in Denmark.

For vital status tracking, patients with DLBCL were linked to the Danish Civil Registration System (CRS) [11] via the unique 10‐digit identifier assigned to all Danish residents at birth or immigration. This identifier is a common data element in all Danish health data systems, thus enabling essentially complete linkage across data systems. The CRS updates vital status and immigration daily for the entire Danish population. The linkage between the DCR and the CRS was used to identify patients with DLBCL who survived 5 years after their initial diagnosis and to track their vital status through December 31, 2023. Vital status follow‐up was nearly complete for DLBCL5yr patients. Only 0.4% emigrated or were lost to follow‐up during our analysis period.

The CRS was also used to create a random general population comparison cohort matched 10:1 to individual DLBCL5yr patients by exact birth year, sex, the calendar year of achieving DLBCL5yr status, and to track their vital status. The random sampling was done with replacement. Sampling with replacement has been shown to be a more valid strategy than random sampling without replacement for mortality analyses comparing patient populations with a general population comparison cohort [12]. For those in the comparison cohort, emigration or loss to follow‐up was minimal (0.4%) during our analysis period.

For patients with DLBCL and the comparison cohort, linkage was implemented with the Danish National Patient Registry (DNPR) [13] and the DCR. The DNPR contains data for all residents in Denmark who have had in‐hospital contact since 1977 and contact with outpatient clinics or emergency departments since 1995. The coding of diagnostic, treatment, and comorbidity data from the DNPR and DCR has been found to be of very high quality in numerous assessments [8].

There were no prior health history restrictions for the comparison cohort in order to provide an accurate counterfactual of general population mortality rates for those who survived 5 years with DLBCL. We used DNPR records from inpatient and outpatient visits, with a lookback period to 1995, to calculate the Charlson Comorbidity Index (CCI) scores for DLBCL patients, DLBCL5yr patients, and their matched members of the comparison cohort [14] (see Table S1 for the ICD‐10 codes used in the CCI.) and to identify other important prevalent comorbidities including hypertension, diabetes, heart disease, chronic lung disease, cerebrovascular disease, and liver disease (see Table S2). Previous research has shown high positive predictive values of ICD‐10 codes in the DNPR for the 19 conditions included in the CCI [15]. The history of a solid tumor was determined from the DCR. We did not include lymphohematopoietic cancers or metastatic solid tumors in the CCI score for DLBCL5yr patients or their comparators to focus on comorbidities that are prevalent in a general population cohort.

### 2.2 Protection of Human Participants

The project was reported to the Danish Data Protection Agency by Aarhus University (record no. 2016–051‐000001/812). All study‐related activities complied with Aarhus University's requirements and Danish laws for protecting the confidentiality and privacy of personal information recorded in health and population registries.

### 2.3 Statistical Analysis

We used Kaplan–Meier methods to plot overall survival (OS) for DLBCL patients and to calculate 1‐, 3‐ and 5‐year OS estimates and the corresponding 95% confidence intervals (CIs).

For the comparative mortality analysis, the index date for DLBCL5yr patients was 5 years after their initial diagnosis date. The index date for members of the comparison cohort was the index date of their matched DLBCL5yr patients. We computed mortality rates per 1000 person‐years at risk (PYRS) for the DLBCL5yr cohort and for the general population comparison cohort overall and stratified by sex, age, calendar period, and cancer stage. We used Cox proportional hazards models to compute matched mortality hazard ratios (HRs) and associated 95% CIs [16, 17] that control implicitly for age, sex, and calendar year. We also used Cox models that controlled additionally for the CCI to compute HRs (with 95% CIs) adjusted for comorbidities (aHR). Further, we evaluated comparative mortality through Cox models stratified by CCI score to examine whether there were different comparative mortality patterns based on the extent of other mortality risk factors. These stratified CCI analyses required controlling for the matching factors in our models because CCI was not a matching factor in our study design. Lastly, we evaluated comparative mortality for specific causes of death other than lymphoma using a matched Cox model as above that controlled for comorbidities.

To test the proportional hazards assumption, we examined log‐negative‐log plots. We did not find evidence of nonproportionality.

## 3 Results

**FIGURE 1:**
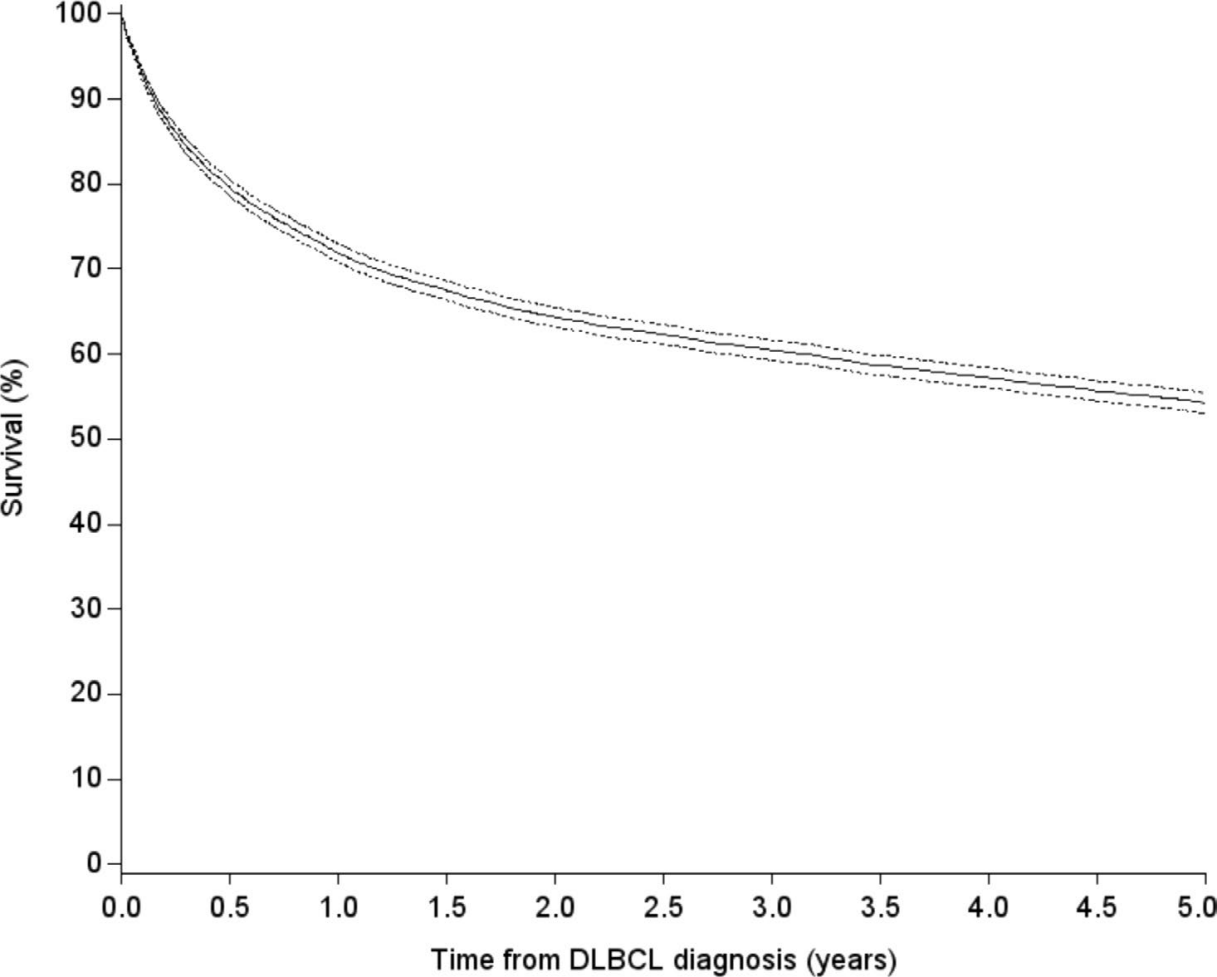
Survival for DLBCL patients diagnosed during 2000–2018.

A total of 7598 patients were diagnosed with DLBCL during the period 2000–2018. The DLBCL5yr cohort included 4164 patients, whereas 3434 patients did not survive 5 years, emigrated, or were lost to follow‐up before 5 years after their initial diagnosis. The proportion of patients who survived 5 years increased during the study period. Overall, the 1‐year OS was 72% (95% CI 71%–73%), the 3‐year OS was 61% (95% CI 60%–62%), and the 5‐year OS was 55% (95% CI 54%–56%) (Figure 1).

As detailed in Table 1, patients who survived 5 years after diagnosis, compared with those who did not survive 5 years, were: younger at the time of DLBCL diagnosis, more likely to have had localized disease, less likely to have been diagnosed during 2000–2004 than during later time periods, and had lower CCI scores and fewer serious prevalent comorbidities. DLBCL5yr patients were also less likely than other patients with DLBCL to have had a history of a solid cancer before their DLBCL diagnosis.

**TABLE 1:** Characteristics of patients diagnosed with DLBCL and patients who survived 5 years and those who survived less than 5 years.

	Diagnosed 2000–2018	%	Survived 5 years	%	Died within 5 years	%
	*N*		*N*		*N*	
Total	7598	100.0	4164	100.0	3434	100.0
Age at diagnosis (year)						
18–49	877	11.5	728	17.5	149	4.3
50–59	1128	14.8	824	19.8	304	8.9
60–69	1989	26.2	1275	30.6	714	20.8
70–79	2207	29.0	1021	24.5	1186	34.5
≥ 80	1397	18.4	316	7.6	1081	31.5
Sex						
Female	3353	44.1	1832	44.0	1521	44.3
Male	4245	55.9	2332	56.0	1913	55.7
Year of diagnosis						
2000–2004	1535	20.2	714	17.1	821	23.9
2005–2009	1892	24.9	1035	24.9	857	25.0
2010–2014	2257	29.7	1277	30.7	980	28.5
2015–2018	1914	25.2	1138	27.3	776	22.6
Stage at diagnosis						
Localized	2171	28.6	1494	35.9	677	19.7
Regional	1136	15.0	656	15.8	480	14.0
Metastatic	2308	30.4	1113	26.7	1195	34.8
Unknown/missing	1983	26.1	901	21.6	1082	31.5
Modified CCI						
0	4122	54.3	2667	64.0	1455	42.4
1	1454	19.1	710	17.1	744	21.7
2	1096	14.4	487	11.7	609	17.7
≥ 3	926	12.2	300	7.2	626	18.2
Solid cancer (before DLBCL diagnosis)	571	7.5	238	5.7	333	9.7
Diabetes (before DLBCL diagnosis)	611	8.0	257	6.2	354	10.3
Hypertension (before DLBCL diagnosis)	1558	20.5	688	16.5	870	25.3
Heart disease (before DLBCL diagnosis)	960	12.6	377	9.1	583	17.0
Chronic lung disease (before DLBCL diagnosis)	656	8.6	256	6.1	400	11.6
Cerebrovascular disease (before DLBCL diagnosis)	540	7.1	183	4.4	357	10.4
Liver disease (before DLBCL diagnosis)	136	1.8	61	1.5	75	2.2

In Table 2, we detail demographic characteristics and comorbidities for the DLBCL5yr cohort and the matched general population comparison cohort as of the DLBCL5yr patients' index dates. Patients in the DLBCL5yr cohort had a higher prevalence of comorbidities, in terms of both the CCI score and the most serious individual comorbidities. In addition, they were somewhat more likely to have had a solid cancer before their 5‐year survival date.

**TABLE 2:** Characteristics of the patients with DLBCL who survived at least 5 years after initial diagnosis and their general population comparison cohort matched by age, sex, and calendar year.

	DLBCL 5‐year survivors	%	Comparison cohort	%
	*N*		*N*	
Total	4164	100.0	41,640	100.0
Age at index date (year)				
< 60	1061	25.5	10,638	25.5
60–69	1088	26.1	10,875	26.1
70–79	1280	30.7	12,757	30.6
≥ 80	735	17.7	7370	17.7
Sex				
Female	1832	44.0	18,320	44.0
Male	2332	56.0	23,320	56.0
Year of index date				
2005–2009	714	17.1	7140	17.1
2010–2014	1035	24.9	10,350	24.9
2015–2019	1277	30.7	12,770	30.7
2020–2023	1138	27.3	11,380	27.3
Modified CCI				
0	1802	43.3	25,139	60.4
1	772	18.5	6606	15.9
2	806	19.4	5540	13.3
≥ 3	784	18.8	4355	10.5
Solid cancer (before index date)	461	11.1	3993	9.6
Diabetes (before index date)	411	9.9	3001	7.2
Hypertension (before index date)	1016	24.4	8907	21.4
Heart disease (before index date)	540	13.0	4942	11.9
Chronic lung disease (before index date)	416	10.0	3294	7.9
Cerebrovascular disease (before index date)	325	7.8	3050	7.3
Liver disease (before index date)	98	2.4	575	1.4

**FIGURE 2:**
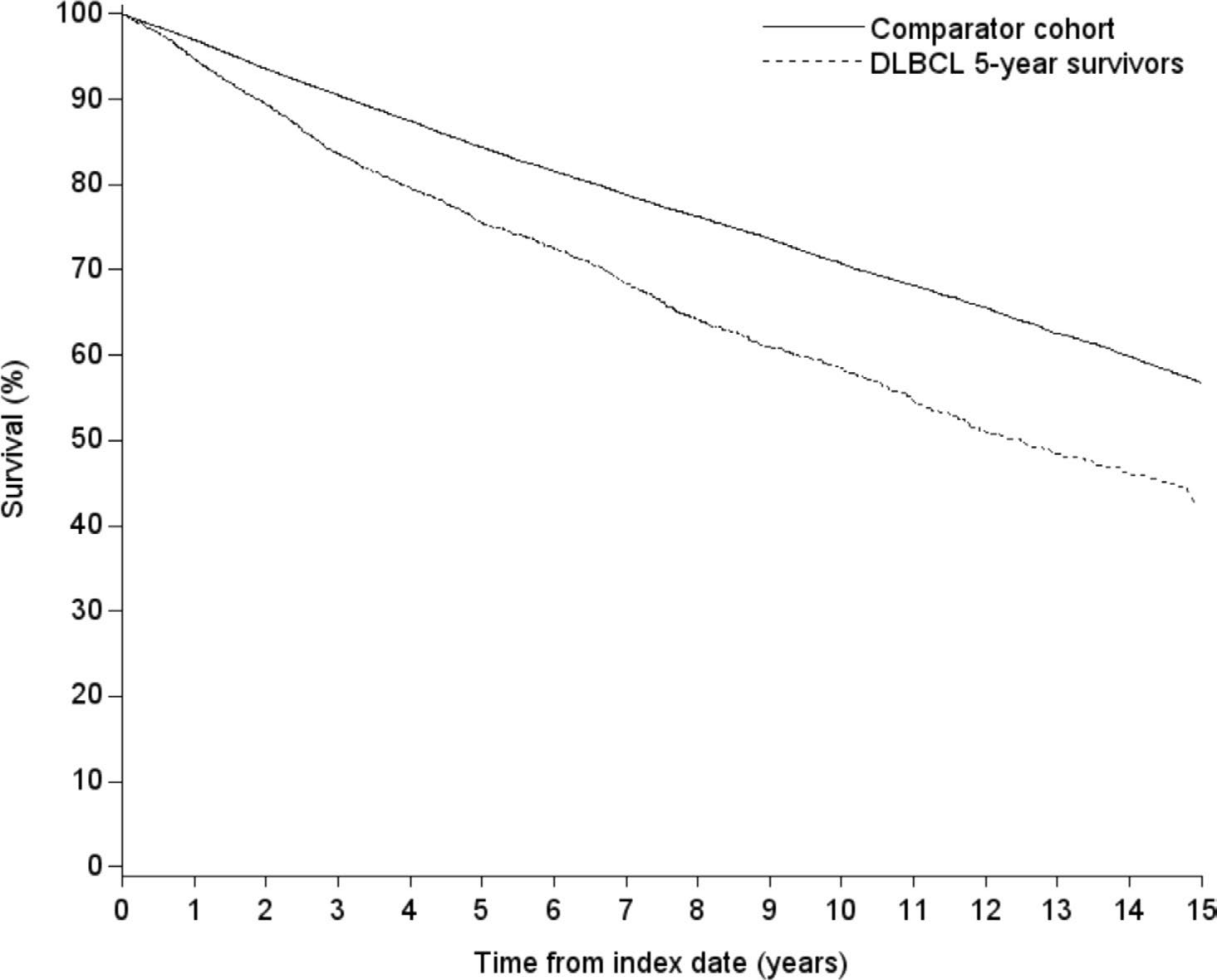
Survival plot for DLBCL 5‐year survivors and the matched comparison cohort.

Table 3 and Figure 2 show higher mortality in the DLBCL5yr cohort than for the matched general population cohort. As shown in Figure 2, the onset of increased mortality was soon after the 5‐year survival date for DLBCL5yr patients and continued for the length of follow‐up. The mortality rate for DLBCL5yr survivors was 56.0 per 1000 PYRS, compared with 35.9 per 1000 PYRS for the general population cohort, an absolute difference of approximately 20 deaths per 1000 PYRS. The absolute differences were approximately 7 deaths per 1000 PYRS for patients < 60 years of age, 23 per 1000 PYRS for patients 60–69 years of age, and > 30 deaths per 1000 PYRS for patients ≥ 70 years of age. Nineteen percent of the DLBCL5yr decedents had lymphoma as their underlying cause of death.

**TABLE 3:** Mortality comparison between patients with DLBCL who survived 5 years after the initial diagnosis and the general population.

	Mortality rate per 1000 PYRS (95% CI) DLBCL 5‐year survivors	Mortality rate per 1000 PYRS (95% CI) comparison cohort	HR (95% CI)	CCI adjusted HR (95% CI)
Overall	56.0 (53.0–58.9)	35.9 (35.2–36.6)	1.7 (1.6–1.8)	1.5 (1.4–1.6)
Age (years)				
< 60	13.0 (10.6–15.4)	6.1 (5.6–6.6)	2.2 (1.8–2.7)	1.4 (1.2–1.8)
60–69	44.3 (39.6–49.1)	20.9 (19.9–21.9)	2.3 (2.0–2.5)	1.9 (1.6–2.1)
70–79	80.0 (73.0–86.9)	49.5 (47.9–51.1)	1.7 (1.6–1.9)	1.5 (1.4–1.7)
≥ 80	174.9 (158.9–190.9)	138.0 (133.8–142.2)	1.3 (1.2–1.5)	1.2 (1.1–1.4)
Sex				
Female	57.8 (53.3–62.2)	35.1 (34.1–36.2)	1.8 (1.7–2.0)	1.6 (1.5–1.7)
Male	54.5 (50.7–58.4)	36.5 (35.5–37.4)	1.6 (1.5–1.7)	1.4 (1.3–1.5)
Stage at diagnosis				
Localized	49.9 (45.5–54.3)	37.0 (35.8–38.1)	1.4 (1.3–1.6)	1.3 (1.2–1.4)
Regional	54.7 (47.8–61.7)	35.4 (33.7–37.0)	1.7 (1.4–1.9)	1.4 (1.2–1.7)
Distant	61.8 (55.8–67.9)	32.7 (31.4–33.9)	2.2 (2.0–2.5)	1.9 (1.7–2.1)
Unknown/missing	62.1 (54.8–69.4)	38.8 (37.1–40.5)	1.7 (1.5–2.0)	1.5 (1.3–1.7)
Calendar period at index date				
2005–2009	58.0 (52.5–63.5)	36.5 (35.2–37.7)	1.9 (1.7–2.1)	1.7 (1.5–1.9)
2010–2014	53.1 (48.4–57.9)	35.1 (34.0–36.3)	1.6 (1.5–1.8)	1.4 (1.3–1.6)
2015–2019	55.3 (49.7–60.9)	37.1 (35.7–38.5)	1.5 (1.4–1.7)	1.3 (1.2–1.5)
2020–2023	62.7 (52.1–73.4)	32.8 (30.4–35.2)	2.1 (1.7–2.5)	1.8 (1.5–2.1)

In terms of relative mortality, the HR for the overall DLBCL5yr cohort was 1.7 (95% CI 1.6–1.8), and the aHR was 1.5 (95% CI 1.4–1.6). We observed variation in the aHRs stratified by age. The age‐specific aHRs were 1.4 (95% CI 1.2–1.8) for patients < 60 years of age; 1.9 (95% CI 1.6–2.1) for patients 60–69 years of age; 1.5 (95% CI 1.4–1.7) for patients 70–79 years of age; and 1.2 (95% CI 1.1–1.4) for patients ≥ 80 years of age. aHRs were fairly similar for men and women.

Mortality was elevated for DLBCL5yr patients for all stages at diagnosis. For those with localized stage, the absolute mortality rate difference was approximately 13 per 1000 PYRS, and the aHR was 1.3 (95% CI 1.2–1.4). For those with distant stage at diagnosis, the mortality rate difference was 29 per 1000 PYRS and the aHR was 1.9 (95% CI 1.7–2.1).

aHRs varied by the calendar period of the index date and were 1.7 (95% CI 1.5–1.9) during 2005–2009; 1.4 (95% CI 1.3–1.6) during 2010–2014; 1.3 (95% CI 1.2–1.5) during 2015–2019; and 1.8 (95% CI 1.5–2.1) during 2020–2023. The trend of decreasing aHRs by calendar periods reversed during the period 2020–2023 that included the COVID‐19 pandemic years. Mortality rates among DLBCL5yr patients were 13% higher in 2020–2023 than in 2015–2019, whereas the mortality in the Danish general population declined 12% between 2015–2019 and 2020–2023. The absolute difference in mortality rates between DLBCL5yr patients and the general population was approximately 65% higher during 2020–2023 than during the two previous calendar periods. Specifically, the absolute difference in mortality rates was 29.9 deaths per 1000 PYRS during the 2020–2023 period as compared with approximately 18 deaths per 1000 PYRS during the two earlier time periods.

In stratified analyses by 4 CCI strata (scores of 0, 1, 2, and ≥ 3), we found the general population cohort to be older than the DLBCL5yr cohort in the three highest CCI categories (averages of 1.7 years older in the CCI = 1 category; 3.2 years older in the CCI = 2 category; and 4.4 years older in the CCI ≥ 3 category). As shown in Table 4, controlling for age, sex, and calendar period, the higher comparative mortality for DLBCL5yr patients was most evident among patients with a CCI score of 0 (i.e., no records of comorbidities in the DNPR that comprise the CCI). The absolute difference in mortality rates was 13.8 deaths per 1000 PYRS, and the HR adjusted for the matching factors was 1.8 (95% CI 1.7–2.0). Mortality differences were smaller in the other CCI strata, presumably reflecting the influence of prevalent comorbidities that increase mortality risk for both populations. For patients with a CCI score of 1, the absolute difference in mortality rates was 4.4 deaths per 1000 PYRS, and the HR adjusted for the matching factors was 1.3 (95% CI 1.1–1.5). For patients with a CCI score of 2, the absolute difference in mortality rates was 6.3 deaths per 1000 PYRS, and the adjusted HR was 1.4 (95% CI 1.2–1.5). Finally, for patients with a CCI score of ≥ 3, the absolute difference in mortality rates was −5.1 deaths per 1000 PYRS, and the adjusted HR was 1.2 (95% CI 1.1–1.3).

**TABLE 4:** Mortality comparison between patients with DLBCL who survived 5 years after the initial diagnosis and the general population comparison cohort, stratified by CCI score.

	Mortality rate (95% CI) DLBCL 5‐year survivors per 1,000 PYRS	Mortality rate (95% CI) comparison cohort per 1,000 PYRS	Crude HR (95% CI)	Matching factors adjusted HR (95% CI)
CCI				
0	34.5 (31.3–37.7)	20.7 (20.0–21.3)	1.7 (1.5–1.9)	1.8 (1.7–2.0)
1	56.7 (49.8–63.6)	52.3 (50.0–54.6)	1.1 (1.0–1.2)	1.3 (1.1–1.5)
2	69.1 (61.5–76.7)	62.8 (59.9–65.6)	1.1 (1.0–1.2)	1.4 (1.2–1.5)
≥ 3	117.4 (105.9–129.0)	122.5 (117.3–127.7)	1.0 (0.9–1.1)	1.2 (1.1–1.3)

Lastly, we evaluated relative mortality controlling for comorbidities for specific causes of death other than lymphoma. Mortality from circulatory diseases was similar for DLBCL5yr patients and for the comparison population (aHR 1.1, 95% CI 1.0, 1.3), including for myocardial infarction (aHR 0.7, 95% CI 0.3–1.3), stroke (aHR 1.1, 95% CI 0.7–1.5), and venous thromboembolism (aHR 1.1, 95% CI 0.4–2.9). Diseases of the respiratory system were elevated for DLBCL5yr patients (aHR 1.4, 95% CI 1.1–1.6), especially for pneumonia (aHR 1.5, 95% CI 1.2–2.0), as was mortality for cancers other than lymphoma (aHR 1.3, 95% CI 1.2, 1.6). Similar analyses restricted to the COVID‐19 years were limited by the small number of deaths. A few disease categories had enough decedents for analysis: all‐cause mortality (aHR 1.9, 95% CI 1.5, 2.4), diseases of the respiratory system (aHR 2.3, 95% CI 1.2, 4.6), and other neoplasms (aHR 1.7, 95% CI 1.0, 2.9).

## 4 Discussion

Our study was designed to determine whether patients with DLBCL who survive 5 years after diagnosis have subsequent mortality rates similar to those of their peers of comparable age and sex. Most previous research on the potential normalization of life expectancy for patients with DLBCL has focused on shorter‐term survivors and subgroups within the DLBCL population, specifically patients who were event‐free for 2 years after the initial diagnosis or patients who were event‐free for 2 years after treatment with a specific chemo‐ or immunotherapy regimen. The present study explored a broader question about comparative survival and included all patients who survived 5 years after the initial diagnosis.

Our findings do not support the hypothesis that mortality among patients with DLBCL who survive 5 years after their initial diagnosis returns to that observed in the general population. The mortality difference between DLBCL5yr patients and their general population peers was estimated to be 20 deaths per 1000 person‐years, and the aHR was 1.5 (95% CI 1.4–1.6).

Five‐year survival for patients with DLBCL increased over most of the study period, and the overall 5‐year survival proportion was 55%. Mortality rates in the DLBCL5yr cohort trended lower from 2005–2009 through 2020, as did HRs adjusted for the CCI. These trends reversed during the calendar period that included the COVID‐19 pandemic. It remains to be determined when mortality among DLBCL5yr survivors will return to pre‐pandemic levels.

The higher mortality over the study period for the DLBCL5yr cohort has several nuances. The increase in relative mortality was lowest for patients ≥ 80 years of age and patients with comorbidities predictive of decreased survival. Conversely, the differences in relative mortality were most apparent against the backdrop of lower mortality due to no comorbidities included in the CCI. In addition to mortality from lymphoma, we also observed increased mortality from respiratory diseases and from cancers other than lymphoma for DLBCL5yr patients.

We note various strengths and limitations of our study design and implementation. Regarding validity issues (i.e., systematic error), the health registry infrastructure in Denmark ensures essentially complete capture of patients with DLBCL and the generation of a random comparison population that is truly representative of the population that gave rise to the DLBCL 5‐year survivors (viz., no referral or selection bias). The infrastructure also provides comparable information on comorbidities among both patients with DLBCL and the comparison population. Lastly, there was only a 0.4% loss to follow‐up for DLBCL5yr patients and the comparison population. These factors support the validity of our results.

The major validity limitation is the potential for residual confounding because of unmeasured or unappreciated confounding factors for DLBCL5yr patients and their general population peers.

Regarding random error considerations, our large study size was sufficient to detect even small mortality differences between the DLBCL5yr cohort and the general population cohort, as evidenced by the very precise 95% confidence intervals, even for subgroup analyses.

In conclusion, we did not find that mortality rates return to general population levels among patients with DLBCL who survive 5 years after diagnosis. Moreover, across all subgroup analyses conducted, mortality was higher for DLBCL5yr patients than their matched general population peers. Increased mortality was evident for DLBCL5yr patients soon after their 5‐year DLBCL survival date. The findings of elevated mortality for DLBCL5yr patients varied in magnitude by age, calendar period, and comorbidity burden and included excess mortality from DLBCL, cancers other than lymphoma, and respiratory diseases. Our findings may have implications for the clinical follow‐up of long‐term survivors. The DLBCL5yr patients have an elevated long‐term mortality, and it will therefore be relevant to follow up the patients clinically for early detection of delayed recurrence, secondary cancer, long‐term side effects of the treatments, and prevention of infections.

## Author Contributions

**John Acquavella:** conceptualization, methodology, interpretation of results, writing, review and editing, supervision, and project administration. **Dóra Körmendiné Farkas:** methodology, data analysis, interpretation of results, validation, writing, review, and editing. **Henrik Toft Sørensen:** conceptualization, methodology, interpretation of results, writing, review, editing, and supervision. All authors contributed to the design of the study, the analysis, the writing of this manuscript, and have reviewed and approved the manuscript's contents.

## Ethics Statement

The project was reported to the Danish Data Protection Agency by Aarhus University (record no. 2016–051‐000001/812). As an observational database study without personal identifiers, this study was judged to be exempt from patient consent requirements and not subject to clinical trial registration. All study‐related activities complied with Aarhus University's requirements and Danish laws for protecting the confidentiality and privacy of personal information recorded in health and population registries.

## Conflicts of Interest

The authors declare no conflicts of interest.

## Supporting information


Table S1.

## Data Availability

The authors are not authorized to make the data used in this study available. Interested parties can make a request from the Danish Data Protection Agency.
