# Supplementary material for: Comparison of Mortality Rates Between Patients With Diffuse Large B Cell Lymphoma Surviving 5 Years After Initial Diagnosis and a Matched General Population Cohort
Source: Cancer Med. 2025 Mar 20;14(6):e70800. doi: 10.1002/cam4.70800 (PMC11924275; doi:10.1002/cam4.70800)
Supplement: Supplementary file 1 — Table S1. [file CAM4-14-e70800-s001.docx]

Supplementary Table 1 – Conditions included in the Charlson Comorbidity Index, according to ICD-10 codes

| Charlson condition | ICD-10 code |
| --- | --- |
| Myocardial infarction | I21, I22, I23 |
| Congestive heart failure | I50, I11.0, I13.0, I13.2 |
| Peripheral vascular disease | I70, I71, I72, I73, I74, I77 |
| Cerebrovascular disease | I60-I69, G45, G46 |
| Dementia | F00-F03, F05.1, G30 |
| Chronic pulmonary disease | J40-J47, J60–J67, J68.4, J70.1, J70.3, J84.1, J92.0, J96.1, J98.2, J98.3 |
| Connective tissue disease | M05, M06, M08, M09, M30, M31, M32, M33, M34, M35, M36, D86 |
| Ulcer disease | K22.1, K25–K28 |
| Mild liver disease | B18, K70.0–K70.3, K70.9, K71, K73, K74, K76.0 |
| Diabetes mellitus | E10.0, E10.1, E10.9, E11.0; E11.1; E11.9 |
| Diabetes mellitus with chronic complications | E10.2–E10.8, E11.2–E11.8 |
| Hemiplegia | G81, G82 |
| Moderate/severe renal disease | I12, I13, N00–N05, N07, N11, N14, N17–N19, Q61 |
| Any tumor | C00–C75 |
| Leukemia | C91–C95 |
| Lymphoma | C81–C85, C88, C90, C96 |
| Moderate/severe liver disease | B15.0, B16.0, B16.2, B19.0, K70.4, K72, K76.6, I85 |
| Metastatic solid tumor | C76–C80 |
| AIDS | B21–B24 |

Supplementary Table 2 – ICD-10 Codes for Comorbidities Included in Tables 1 and 2

| Comorbidity | ICD-10 codes |
| --- | --- |
| Hypertension | I10-I16 |
| Diabetes mellitus | E08-E13 |
| Heart disease | I20-I25 |
| Solid cancers | C00-C75 |
| Chronic lung diseases | J40-J47 |
| Cerebrovascular disease | I60-I69 |
| Liver disease | K70-K76 |
